# Gendered role assignment and musical self-efficacy in extracurricular music education: hidden curriculum pathways to inclusion and equity

**DOI:** 10.3389/fpsyg.2026.1888955

**Published:** 2026-08-03

**Authors:** Keran Li, Jinglei Zhao, Mo Zhou

**Affiliations:** 1 Faculty of Music and Dance, Liaocheng University, Liaocheng, China; 2 Liaocheng Vocational and Technical College, Liaocheng, Shandong, China; 3 School of Culture, Tourism, Health and Education, Dongying Vocational College, Dongying, Shandong, China; 4 Hunan Normal University, Changsha, China

**Keywords:** conditional indirect association, extracurricular music education, gender schema internalization, hidden curriculum, musical self-efficacy, perceived role assignment, perceived teacher fairness

## Abstract

Gender-based disparities in musical role assignment and self-efficacy have been documented across educational contexts, yet the association between perceived role allocation and self-evaluative beliefs remains insufficiently specified in extracurricular music settings. Using cross-sectional survey data from 312 university students enrolled in extracurricular music classes, this study examined whether gender schema internalization (GSI) statistically accounted for part of the association between perceived role assignment (PRA) and musical self-efficacy (MSE), and whether this indirect association differed by gender. Female students reported lower perceived role assignment, less equitable teacher and peer climate perceptions, higher gender schema internalization, and lower musical self-efficacy. Bootstrap analyses indicated a significant indirect association from PRA to MSE through GSI [*B* = 0.098, 95% CI (0.051, 0.152)], and a stronger conditional indirect effect among female than male students (female *B* = 0.105, male *B* = 0.038). A supplementary serial model suggested that perceived teacher fairness was linked to self-efficacy primarily through role-allocation perceptions and, secondarily, through the sequential PRA-GSI chain, whereas the direct TBP-GSI-MSE path was weak. These findings are consistent with the interpretation that gendered role perceptions may become linked to self-evaluative beliefs partly through internalized gender-role schemas. Because the study relied on cross-sectional self-report data, the findings should be interpreted as association patterns rather than evidence of causal sequence.

## 1 Introduction

Gender based disparities in role assignment and performance opportunity represent one of the most persistent yet understudied structural features of music education environments. Across decades of research and multiple national contexts, empirical studies have consistently documented that the instruments students choose, the performance roles they are assigned, and the degree of leadership they are afforded in ensemble and classroom settings are patterned by gender (Hallam et al., 2008, 2020; McPherson et al., 2015). Female students are more often directed toward accompaniment, supportive, and background roles, whereas lead performance positions, such as soloist designations, section leadership, and featured ensemble parts, have more often been associated with male students (Borgström Källén and Ferm Almqvist, 2025; Green and Mitchell, 2025). These patterns appear early in musical training and remain visible across educational levels, as Hallam et al. (2020) showed in a large-scale study of 3,325 young instrumentalists spanning beginner to Grade 8 level, where gender differences in motivation, perceived competence, and performance aspiration were evident at each stage of expertise. Yet the psychological interpretations students attach to such structural inequalities remain less clearly understood, particularly in extracurricular music contexts where participation is nominally voluntary and is therefore often framed as purely interest-driven (Knapp, 2021; Nonte et al., 2022). Jackson’s (1968) concept of the hidden curriculum draws attention to the implicit norms, expectations, and behavioral codes that circulate alongside formal educational content; applied to music settings, this perspective suggests that repeated assignment of lead roles to male students may communicate informal messages about who is visible, who is capable, and who belongs at the front (Donovan, 2023; Rossouw and Frick, 2023; Sánchez and Barber, 2020). Bem’s (1981) gender schema theory offers a useful framework for interpreting how such cues may become self-referential, proposing that repeated exposure to gender-organized environments can shape the cognitive schemas through which self-relevant information is interpreted. In this sense, a student who repeatedly perceives herself as excluded from lead performance roles may become more likely to understand that pattern through a gendered lens, such that it becomes relevant to her sense of musical identity and competence (Bem, 1981; Kollmayer et al., 2018; Li et al., 2022). Extracurricular music settings are therefore analytically important not because they can be assumed *a priori* to intensify these processes, but because the discourse of free choice and individual passion may make structurally patterned inequalities harder for participants to name and contest (Kelley, 2021; Knapp, 2021; Kuuse and Westerlund, 2022). Teacher behavior and peer attitudes may matter here not only as sources of external feedback, but also as contextual cues through which students interpret themselves within gendered learning environments. Social role theory offers a complementary perspective, suggesting that gendered socialization may shape how strongly such cues are noticed and incorporated into self-evaluative processes (Eagly and Wood, 2016).

Beyond explicit instruction, students’ self-perceptions are also shaped by repeated social signals regarding who is encouraged, recognised, and expected to succeed in particular musical roles. Teacher expectations and classroom interactions can function as important sources of competence-related information, influencing students’ perceptions of ability, belonging, and future possibilities (Inan-Kaya and Rubie-Davies, 2022; Rubie-Davies and Hattie, 2025). Within music education contexts, these processes may be particularly important because opportunities for visibility, leadership, and performance exposure are closely connected with students’ developing musical identities and confidence. Previous research suggests that musical self-efficacy is not formed solely through individual ability or achievement, but also through contextual experiences, including instructional feedback, social support, meaningful opportunities for participation, and performance-related experiences (Gill et al., 2023; Zarza-Alzugaray et al., 2020; MacAfee and Comeau, 2020). Gendered expectations may further influence these processes by shaping how students interpret role opportunities and evaluate their own competence, as gender-related beliefs and social expectations can influence musical identity formation, participation patterns, and self-evaluative processes (O’Neill, 1997; Timmermans and Rubie-Davies, 2023). Recent research in music education further highlights that gender inequalities may persist through institutional cultures, implicit expectations, and unequal recognition of students’ capabilities within higher music education environments (MacGregor, 2024). Moreover, recent meta-analytic evidence reinforces the importance of self-efficacy beliefs in understanding music performance-related psychological processes and outcomes (Zelenak, 2024).

One important set of outcomes potentially associated with this interpretive process concerns musical self-efficacy and, secondarily in the present study, sense of agency. Musical self-efficacy, defined as the domain-specific belief in one’s capacity to successfully execute musical learning and performance tasks, has been consistently linked to practice persistence, performance quality, and willingness to assume visible or demanding roles in ensemble contexts (Ritchie and Williamon, 2011; McPherson and McCormick, 2006; McPherson et al., 2015). Research further suggests that self-efficacy beliefs in music are multidimensional, with performance self-efficacy showing particularly pronounced gender differences in contexts involving public visibility and evaluative scrutiny (Casanova et al., 2023; Ritchie and Williamon, 2011). Across prior studies, female students have often reported lower musical self-efficacy than male peers, and this difference appears especially pronounced in settings tied to leadership, visibility, and performance opportunity (Hallam et al., 2020; Wang and Yu, 2023). Related work in adjacent domains likewise suggests that internalized gender-role beliefs may be associated with lower efficacy-related outcomes among girls and young women (Luo and Wang, 2022). Despite this converging evidence, at least three gaps remain in the literature. First, few studies in music education have directly modeled whether the association between perceived role assignment and self-efficacy is statistically conveyed through gender schema internalization. Second, although teacher behavior has been widely studied as a source of differential expectations and gendered feedback, its place as an upstream contextual correlate within such an association pattern has not been clearly tested in extracurricular music contexts. Third, the extent to which gender may shape the strength of this internalization-related association remains uncertain. Addressing these gaps is the purpose of the current study. Drawing on gender schema theory (Bem, 1981), hidden curriculum theory (Jackson, 1968), and social cognitive perspectives on self-efficacy development (Bandura, 1997), the present study used cross-sectional survey data from 312 university students enrolled in extracurricular music classes to test whether perceived role assignment was associated with musical self-efficacy through gender schema internalization, whether perceived teacher behavior contributed to this pattern through role-allocation perceptions, and whether the indirect association varied by gender. Sense of agency was retained as an additional outcome for descriptive comparison, but the focal explanatory models centered on musical self-efficacy because it was the more theoretically specified endpoint in the present design. We hypothesized that female students would report lower perceived role assignment, less equitable teacher behavior perception, and less equitable peer attitude perception, alongside higher gender schema internalization and lower musical self-efficacy and sense of agency than male students (H1, H2); that gender schema internalization would partially mediate the association between perceived role assignment and musical self-efficacy (H3); that teacher behavior perception would be indirectly associated with musical self-efficacy through perceived role assignment and gender schema internalization (H4); and that the indirect association between perceived role assignment and musical self-efficacy via gender schema internalization would be stronger among female students than among male students (H5, H6).

## 2 Methods

### 2.1 Research design and participants

This study employed a cross-sectional survey design to examine psychological associations linking gendered role assignment in extracurricular music settings with students’ musical self-efficacy and sense of agency. Participants were university students aged 18–22 years (M = 19.93, SD = 1.12) who were currently enrolled in at least one extracurricular music class at the time of data collection. A total of 312 participants were recruited through convenience sampling across multiple university campuses, comprising 178 female (57.1%), 127 male (40.7%), and 7 non-binary or gender-diverse (2.2%) students. Participation was voluntary, anonymous, and confidential, and informed consent was obtained before questionnaire completion. Participants identifying as non-binary or another gender category were retained in descriptive reporting. However, because this subgroup size was too small to support reliable inferential estimation, inferential analyses were restricted to female and male participants. This decision reflects a sample-size limitation rather than any assumption regarding the substantive importance of gender-diverse students; future studies should use targeted recruitment and more inclusive analytic designs to represent gender-diverse students more adequately. Inclusion criteria required active enrollment in an extracurricular music course, with no restrictions placed on instrument type or years of prior study.

The study protocol involving human participants was reviewed and approved by the Ethics Committee of Xihua University (Approval No. XHLL202503-01; approval date: March 31, 2025). The questionnaire introduction explained the purpose of the study, the voluntary nature of participation, the right to withdraw before submission, and the procedures used to protect anonymity and confidentiality.

### 2.2 Measures

All items were rated on a 5-point Likert scale ranging from 1 (strongly disagree) to 5 (strongly agree). Unless otherwise noted, higher scores indicated stronger endorsement of the focal construct. For the contextual climate measures, negatively worded items were reverse-coded so that higher TBP and PAP scores reflected more equitable and less gender-biased perceived classroom environments. All scales were adapted from existing validated instruments and modified to fit the context of extracurricular music classes. Reliability and validity indices for all measures are reported in Table 1. Because several constructs were adapted to a specific extracurricular music context, the representative items below are provided to clarify the conceptual focus of each scale; the adapted item wording should be considered when interpreting construct distinctiveness.

**Table 1:** Scale reliability and confirmatory factor analysis fit indices.

Scale	Items	Reliability			CFA fit				
		*α*	*ω*	AVE	*χ^2^/df*	CFI	RMSEA	[90% CI]	SRMR
PRA	6	0.852	0.852	0.490	1.68	0.991	0.047	[0.027, 0.067]	0.025
TBP	8	0.892	0.892	0.509	0.99	1.000	0.000	[0.000, 0.020]	0.020
PAP	6	0.852	0.853	0.493	1.07	0.999	0.015	[0.000, 0.035]	0.020
GSI	7	0.868	0.868	0.486	1.27	0.995	0.030	[0.010, 0.050]	0.022
MSE	8	0.905	0.905	0.545	0.59	1.007	0.000	[0.000, 0.020]	0.015
SoA	6	0.852	0.852	0.491	1.06	0.999	0.014	[0.000, 0.034]	0.019

*α* = Cronbach’s alpha; *ω* = McDonald’s omega; AVE = average variance extracted. All CFA models were re-estimated from the present dataset using a one-factor specification for each scale. RMSEA confidence intervals are reported as 90% intervals. All factor loadings *p* < 0.001.

#### 2.2.1 Perceived role assignment (PRA)

Six items assessed the extent to which participants perceived themselves as being assigned lead performance roles (e.g., soloist, section leader, featured performer) within their music class. Items were adapted from role perception scales used in music education research (e.g., “In my music class, I am frequently chosen to perform lead parts”). Internal consistency was acceptable (*α* = 0.852, *ω* = 0.875).

#### 2.2.2 Teacher behavior perception (TBP)

Eight items assessed students’ perceptions of the extent to which their music teacher behaved in equitable rather than gender-differentiated ways when allocating roles and providing feedback. The item pool included both negatively worded indicators of gendered expectations and positively worded indicators of equitable treatment; negatively worded items were reverse-coded so that higher scores reflected greater perceived teacher fairness and less perceived gender bias. A representative positively scored item was, “My teacher gives students of different genders similar opportunities to take on challenging musical parts.” Items were adapted from the teacher expectancy and classroom climate literature. Internal consistency was good (*a* = 0.892, omega = 0.911).

#### 2.2.3 Peer attitude perception (PAP)

Six items assessed students’ perceptions of classmates’ gender-related attitudes regarding performance roles. As with TBP, the item pool included both negatively and positively worded statements, with scoring oriented so that higher values reflected a more equitable and less gender-stereotyped peer climate. A representative positively scored item was, “Students in my class are open to seeing any gender take on lead roles.” Items were adapted from peer influence scales in social psychology. In the present study, PAP was included as a supplementary contextual variable to capture the broader peer climate of extracurricular music settings. It was used primarily for descriptive and correlational purposes rather than incorporated into the core indirect-association pathway, which centered more specifically on teacher-related role allocation processes. Internal consistency was acceptable (*a* = 0.852, omega = 0.866).

#### 2.2.4 Gender schema internalization (GSI)

Seven items measured endorsement of gender-differentiated beliefs about musical performance roles, treated here as an indicator of internalized schema salience rather than as an exhaustive measure of identity structure. A representative item was, “I believe certain musical roles are more suitable for my gender.” Items were adapted from gender schema assessment instruments (Bem, 1981) and modified for the music education context. Internal consistency was good (*a* = 0.868, omega = 0.898).

#### 2.2.5 Musical self-efficacy (MSE)

Eight items assessed participants’ confidence in their ability to perform musical tasks at a high level and to take on lead performance roles. The scale was intended to capture perceived capability rather than actual access to opportunities, even though those two experiences may be empirically related in practice. A representative item was, “I am confident in my ability to perform as the lead musician in an ensemble.” Items were adapted from Bandura’s (1997) self-efficacy framework and domain-specific music performance confidence scales. Internal consistency was excellent (*a* = 0.905, omega = 0.923). Conceptually, MSE was distinguished from PRA because PRA referred to perceived allocation of roles by the learning environment, whereas MSE referred to perceived personal capability; nevertheless, the shared focus on visible or lead performance roles means that partial item-content overlap remains possible and should be considered when interpreting associations between the two constructs.

#### 2.2.6 Sense of agency (SoA)

Six items measured the extent to which participants felt they had autonomy and active influence over their musical learning and performance opportunities. In the present study, SoA was treated as a descriptively important secondary outcome rather than as the focal endpoint in the indirect-association models. A representative item was, “I feel I have a say in which musical roles I take on in class.” Items were adapted from agency scales in educational psychology. Internal consistency was acceptable (*a* = 0.851, omega = 0.879).

### 2.3 Data analysis

All analyses were conducted in Python (version 3.11). Descriptive statistics, reliability analyses, correlational analyses, and regression-based models were implemented using standard analytical libraries, including NumPy (version 1.24), SciPy (version 1.11), and Pandas (version 2.0). Prior to hypothesis testing, confirmatory factor analyses (CFA) were conducted for each scale to evaluate measurement model fit using the semopy library (version 2.3; Igolkina and Meshcheryakov, 2019), with parameters estimated via maximum likelihood (ML) estimation. All CFA models were specified as unidimensional, with item residuals constrained to be uncorrelated; no post-hoc residual covariances were freed in the final solutions. Model adequacy was assessed using conventional fit criteria, including the comparative fit index (CFI), root mean square error of approximation (RMSEA) with 90% confidence intervals, and standardized root mean square residual (SRMR), with CFI values above 0.95, RMSEA values below 0.08, and SRMR values below 0.06 taken to indicate acceptable fit (Hu and Bentler, 1999).

Descriptive statistics and independent-samples *t*-tests were computed to assess gender differences across study variables (H1 and H2), with Cohen’s *d* reported as an index of effect size. The simple indirect-association model (H3) and serial indirect-association model (H4) were estimated using ordinary least squares regression with bootstrap-based confidence intervals based on 5,000 resamples (Hayes, 2022), with confidence intervals constructed using the percentile method. Indirect effects were considered statistically significant when the 95% bootstrap confidence interval excluded zero. Because the data were cross-sectional, these models were interpreted as tests of indirect associations rather than evidence of temporal or causal mediation. Hierarchical multiple regression was used for the exploratory moderation analysis (H5), with the predictor mean-centered before computing the interaction term. The conditional indirect association (H6) was evaluated using Hayes’s (2015) index of moderated mediation as a statistical index of whether the indirect association differed by gender. Age was included in Model A. To address potential confounding by musical background, Model B added instrument type and years of musical study as covariates. Additional reviewer-requested diagnostics included multigroup measurement invariance tests across gender, Harman’s single-factor test and a single-factor CFA for common method variance, HTMT estimates, and a PRA-MSE two-factor versus one-factor CFA comparison for discriminant validity. Because the non-binary/gender-diverse subgroup was too small to support stable inferential estimation, inferential analyses were limited to female and male participants.

## 3 Empirical results

### 3.1 Sample characteristics and descriptive statistics

Table 2 presents the demographic characteristics of the full sample. The three gender groups were similar in age (M range = 19.91–20.43 years). Instrument distribution showed a gendered pattern, with female participants more frequently represented in piano and strings, and male participants more frequently represented in wind/brass and percussion. Female participants also reported slightly longer cumulative study than male participants (>6 years: 29.8% vs. 26.8%). These differences are substantively important because instrument type and prior musical experience may shape access to solo, leadership, or featured roles as well as confidence in performance. Accordingly, the observed gender contrasts should be interpreted with caution until future analyses can control instrument category, years of study, and other indicators of performance history.

**Table 2:** Demographic characteristics of participants (*N* = 312).

Variable	Female (*n* = 178)	Male (*n* = 127)	Non-binary (*n* = 7)	Total (*N* = 312)
Age				
M (SD)	19.91 (1.05)	19.91 (1.20)	20.43 (1.09)	19.92 (1.11)
Range	18–22	18–22	18–22	18–22
Primary instrument, *n* (%)				
Piano	73 (41.0)	24 (18.9)	1 (14.3)	98 (31.4)
Strings	41 (23.0)	23 (18.1)	3 (42.9)	67 (21.5)
Wind/brass	22 (12.4)	41 (32.3)	3 (42.9)	66 (21.2)
Percussion	8 (4.5)	28 (22.0)	0 (0.0)	36 (11.5)
Voice/Other	34 (19.1)	11 (8.7)	0 (0.0)	45 (14.4)
Years of study, *n* (%)				
1–3 years	44 (24.7)	53 (41.7)	2 (28.6)	99 (31.7)
4–6 years	81 (45.5)	40 (31.5)	4 (57.1)	125 (40.1)
>6 years	53 (29.8)	34 (26.8)	1 (14.3)	88 (28.2)

Non-binary/other group excluded from inferential analyses due to small *n*.

Independent-samples *t*-tests revealed significant gender differences across all six study variables (see Table 3). Female participants reported significantly lower perceived role assignment than male participants (M = 3.27 vs. 3.74, *d* = −0.71), indicating that female students in this sample perceived themselves as being assigned lead roles less frequently in music class contexts. Female participants also reported lower teacher behavior perception (*d* = −0.39) and peer attitude perception (*d* = −0.50). Given the scoring of these scales, lower values indicate less equitable teacher and peer climates and therefore less favorable perceptions of the gendered role environment across both instructional and peer dimensions. In contrast, female participants scored significantly higher on gender schema internalization than male participants (M = 3.68 vs. 3.32, *d* = 0.51), indicating stronger endorsement of gender-differentiated role beliefs. Female participants also reported significantly lower musical self-efficacy (M = 3.24 vs. 3.81, *d* = −0.73) and sense of agency (M = 3.11 vs. 3.78, *d* = −0.76), with effect sizes in the medium-to-large range. Taken together, these findings support H1 and H2 and indicate that gender differences in perceived role assignment, contextual climate, and psychological outcomes were evident in this sample of students enrolled in extracurricular music settings.

**Table 3:** Descriptive statistics and gender differences on study variables.

Variable	Female (*n* = 178)		Male (*n* = 127)		*t*	*df*	*p*	Cohen’s *d*
	M	SD	M	SD				
PRA	3.27	0.64	3.74	0.70	−6.10	303	<0.001	−0.71
TBP	3.40	0.72	3.68	0.75	−3.32	303	0.001	−0.39
PAP	3.36	0.76	3.72	0.67	−4.29	303	<0.001	−0.50
GSI	3.68	0.72	3.32	0.66	4.40	303	<0.001	0.51
MSE	3.24	0.82	3.81	0.70	−6.31	303	<0.001	−0.73
SoA	3.11	0.93	3.78	0.78	−6.58	303	<0.001	−0.76

PRA, perceived role assignment; TBP, teacher behavior perception; PAP, peer attitude perception; GSI, gender schema internalization; MSE, musical self-efficacy; SoA, sense of agency. All scales 1–5. ^**^p < 0.01, ^***^p < 0.001.

### 3.2 Scale reliability and measurement validity

Prior to hypothesis testing, the psychometric properties of all six scales were evaluated. As shown in Table 1, internal consistency was acceptable to excellent across scales, with Cronbach’s alpha ranging from 0.851 to 0.905 and McDonald’s omega ranging from 0.852 to 0.923. One-factor CFA models also showed acceptable fit across scales, suggesting that the adapted measures were internally coherent in the present sample. Because gender comparison is central to the study, a multigroup CFA was further conducted for the focal measurement model including PRA, GSI, and MSE. The configural model showed acceptable fit, chi-square (369) = 417.38, CFI = 0.981, RMSEA = 0.021. Metric invariance was supported, chi-square (387) = 430.11, CFI = 0.983, RMSEA = 0.019, absolute Delta CFI = 0.002, absolute Delta RMSEA = 0.002, Delta chi-square (18) = 12.73, *p* = 0.807. Scalar invariance was also supported, chi-square (408) = 457.03, CFI = 0.981, RMSEA = 0.020, absolute Delta CFI = 0.002, absolute Delta RMSEA = 0.001, Delta chi-square (21) = 26.92, *p* = 0.174. Because Delta CFI was below 0.010 and Delta RMSEA was below 0.015 at both the metric and scalar steps, measurement equivalence across gender was established.

Table 4 presents the means, standard deviations, and intercorrelations among all study variables. Perceived role assignment (PRA) was positively associated with both contextual climate variables (TBP: *r* = 0.32; PAP: *r* = 0.21) and both outcome variables (MSE: *r* = 0.40; SoA: *r* = 0.35), and was negatively associated with gender schema internalization (GSI: *r* = −0.35). GSI was negatively associated with MSE (*r* = −0.34) and SoA (*r* = −0.39), whereas MSE and SoA were moderately correlated (*r* = 0.52). Discriminant validity was further evaluated using HTMT. The focal PRA-MSE HTMT value was 0.392, well below the conservative 0.85 criterion; all HTMT values among the study constructs were below 0.85, with the largest value being 0.557. In addition, a two-factor CFA separating PRA and MSE fit the data very well, chi-square (76) = 79.06, CFI = 0.998, RMSEA = 0.012, and fit substantially better than a one-factor model, chi-square (77) = 608.09, CFI = 0.718, RMSEA = 0.151, Delta chi-square (1) = 529.03, *p* < 0.001. These findings support the empirical distinction between perceived role assignment and musical self-efficacy despite their shared reference to lead or visible performance roles. Common method variance was also examined. Harman’s single-factor test showed that the first unrotated factor explained 22.21% of the total item variance, below the common 40% threshold. A single-factor CFA for all measured items showed poor fit, chi-square (779) = 3986.64, chi-square/*df* = 5.12, CFI = 0.399, RMSEA = 0.116. These diagnostics suggest that common method variance was unlikely to fully account for the observed associations.

**Table 4:** Pearson correlation matrix, means, and standard deviations (*N* = 305).

Variable	M	SD	1	2	3	4	5	6
1. PRA	3.47	0.71	—					
2. TBP	3.51	0.75	0.32^***^	—				
3. PAP	3.51	0.74	0.21^***^	0.43^***^	—			
4. GSI	3.53	0.72	−0.35^***^	−0.13^*^	−0.07	—		
5. MSE	3.48	0.82	0.40^***^	0.17^**^	0.14^*^	−0.34^***^	—	
6. SoA	3.39	0.93	0.35^***^	0.18^**^	0.11	−0.39^***^	0.52^***^	—

^*^p < 0.05, ^**^p < 0.01, ^***^p < 0.001.

### 3.3 Indirect association involving gender schema internalization

To test H3, a simple indirect-association model was estimated with PRA as the predictor, GSI as the statistical mediator, and MSE as the outcome, controlling for age. As shown in Table 5, PRA was significantly negatively associated with GSI (*a* path: *B* = −0.368, *β* = −0.363, *p* < 0.001), indicating that students who perceived fewer lead-role assignments tended to report higher levels of gender schema internalization. GSI was, in turn, significantly negatively associated with MSE (*b* path: *B* = −0.265, *β* = −0.232, *p* < 0.001), such that stronger gender schema internalization was associated with lower musical self-efficacy. The direct association between PRA and MSE remained significant after accounting for GSI (*c*′ path: *B* = 0.362, *β* = 0.312, *p* < 0.001), indicating that GSI statistically accounted for part, but not all, of the PRA-MSE association. The bootstrap-based indirect effect was significant and positive [*B* = 0.098, 95% CI (0.051, 0.152)], with the confidence interval excluding zero, representing 21.2% of the total association. These results support H3 and are consistent with, but do not by themselves establish, a model in which lower perceived role assignment is associated with stronger gender schema internalization, which in turn is associated with lower musical self-efficacy. Robustness analyses showed that the focal indirect association remained substantively unchanged after adding instrument type and years of musical study. In Model A, the age-adjusted indirect effect was *B* = 0.098, 95% CI [0.050, 0.151]. In Model B, after controlling for instrument type and years of musical study, the *a* path remained significant (*B* = −0.351, *p* < 0.001), the *b* path remained significant (*B* = −0.253, *p* < 0.001), and the indirect effect remained significant [*B* = 0.089, 95% CI (0.042, 0.142)]. The indirect-effect estimate changed only modestly from 0.098 to 0.089, indicating that the result was not driven by instrument type or years of musical study.

**Table 5:** Indirect association: gender schema internalization statistically accounting for PRA → musical self-efficacy.

Path	*B*	SE	*β*	*t*	*p*	95% CI
Outcome: GSI (*R*^2^ = 0.135)						
*a* PRA → GSI	−0.368	0.055	−0.363	−6.74	<0.001	—
Outcome: MSE (*R*^2^ = 0.206)						
*c*′ PRA → MSE (direct)	0.362	0.064	0.312	5.64	<0.001	—
*b* GSI → MSE	−0.265	0.063	−0.232	−4.20	<0.001	—
Indirect effect (Bootstrap 5,000 resamples)						
PRA → GSI → MSE	0.098	0.026	—	—	—	[0.051, 0.152]
Mediation ratio = 21.2% | CI excludes zero, confirming significant partial mediation.						

Age included as covariate. CI excludes zero, indicating a statistically non-zero indirect association.

To test H4, a serial indirect-association model was estimated with TBP as the upstream predictor, PRA and GSI as sequential statistical mediators, and MSE as the outcome. As shown in Table 6, TBP was significantly associated with MSE at the total-effect level (*B* = 0.180), while the direct association after inclusion of both mediators was reduced to non-significance (*B* = 0.043). Three indirect pathways were examined. Path 1 (TBP → PRA → MSE) was positive and significant [*B* = 0.102, 95% CI (0.053, 0.161)], indicating that more equitable perceived teacher behavior was associated with higher musical self-efficacy through more favorable role-allocation perceptions. Path 2 (TBP → GSI → MSE) was comparatively small [*B* = 0.007, 95% CI (−0.021, 0.036)] and did not show a clearly non-zero bootstrap interval. Path 3 (TBP → PRA → GSI → MSE) was positive and significant [*B* = 0.028, 95% CI (0.013, 0.048)], suggesting an additional sequential association through both perceived role assignment and gender schema internalization. Taken together, these findings support H4 only in a restrained sense: perceived teacher fairness appears related to self-efficacy most clearly through role-allocation perceptions, with more limited evidence for a distinct direct TBP-GSI-MSE pathway.

**Table 6:** Serial indirect association: TBP → PRA → GSI → MSE.

Effect	*B*	Bootstrap 95% CI	% of total
Total effect TBP → MSE	0.180	—	—
Direct effect (controlling PRA, GSI)	0.043	—	—
Path 1: TBP → PRA → MSE	0.102	[0.053, 0.161]	—
Path 2: TBP → GSI → MSE	0.007	[−0.021, 0.036]	4.1%
Path 3: TBP → PRA → GSI → MSE	0.028	[0.013, 0.048]	15.5%

Bootstrap resamples = 5,000. Path 1 and Path 3 confidence intervals exclude zero. The TBP → GSI → MSE path is weak and includes zero in the bootstrap interval, so it should be interpreted cautiously.

### 3.4 Gender as a moderator of the indirect pathway

To examine H5, an exploratory hierarchical regression was conducted with MSE as the outcome, entering PRA, gender, and age in Step 1, and the PRA × Gender interaction term in Step 2. As shown in Table 7, Step 1 accounted for significant variance in MSE (*R*^2^ = 0.209, *p* < 0.001), with both PRA (*B* = 0.368, *p* < 0.001) and gender (*B* = 0.392, *p* < 0.001) emerging as significant predictors. The addition of the interaction term in Step 2 did not significantly improve model fit (*ΔR*^2^ = 0.002, *p* = 0.438), and the PRA × Gender interaction was non-significant (*B* = −0.098, *p* = 0.438), indicating that the direct association between perceived role assignment and musical self-efficacy did not differ significantly by gender in this sample. Thus, the exploratory moderation test did not provide support for H5. Nonetheless, simple slope analyses showed that the association between PRA and MSE was positive and significant for both female (*B* = 0.421, *p* < 0.001) and male participants (*B* = 0.305, *p* = 0.001). The absence of a significant interaction on the direct pathway does not rule out the possibility that gender differences may emerge in the indirect association, which was examined in the subsequent conditional indirect-association analysis.

**Table 7:** Moderation analysis: gender moderating PRA → MSE.

Predictor	*B*	SE	*t*	*p*	*ΔR* ^2^
Step 1 (*R*^2^ = 0.209)					
PRA	0.368	0.063	5.81	<0.001	0.209^***^
Gender (0 = F, 1 = M)	0.392	0.090	4.34	<0.001	
Age	0.024	0.038	0.64	0.522	
Step 2 (*R*^2^ = 0.211)					
PRA × Gender	−0.098	0.126	−0.78	0.438	0.002
Simple slopes					
PRA → MSE (female)	0.421	—	4.61	<0.001	—
PRA → MSE (male)	0.305	—	3.60	<0.001	—

PRA mean-centered. The interaction was non-significant, indicating that any gender difference observed in this analysis was concentrated in the indirect association rather than in the direct PRA → MSE slope. ***p < 0.001.

To test H6, a moderated indirect-association model was estimated in which gender was specified as a moderator of the *a* path (PRA → GSI), with GSI statistically accounting for part of the association between PRA and MSE. As shown in Table 8, the conditional indirect-association index was non-zero and negative [IMM = −0.068, 95% CI (−0.133, −0.016)], reflecting the coding of gender (0 = female, 1 = male) and indicating that the indirect association was stronger among female students. Examination of the conditional indirect effects revealed that the indirect pathway was significant for both female [*B* = 0.105, 95% CI (0.047, 0.173)] and male participants [*B* = 0.038, 95% CI (0.001, 0.083)]; however, the magnitude of the indirect effect was larger among female participants. This difference was also reflected in the gender-stratified a-path estimates, with the association between PRA and GSI being stronger for female students (*B* = −0.455) than for male students (*B* = −0.162). The conditional indirect-association model accounted for 17.1% of the variance in GSI and 24.4% of the variance in MSE. Overall, these findings suggest that the indirect association between perceived role assignment and musical self-efficacy via gender schema internalization was stronger among female students than among male students in this sample. The moderated indirect association also remained stable in the covariate-adjusted robustness model. In Model A, the index was *B* = −0.068, 95% CI [−0.131, −0.015]. In Model B, after controlling for instrument type and years of musical study, the index remained significant [*B* = −0.062, 95% CI (−0.125, −0.010)]. The conditional indirect effects also remained significant for female students [*B* = 0.103, 95% CI (0.044, 0.169)] and male students [*B* = 0.041, 95% CI (0.006, 0.087)]. Results remained substantively unchanged after controlling for instrument type and years of musical study.

**Table 8:** Conditional indirect association: gender moderating the PRA → GSI → MSE pathway.

Effect	*B*	SE	Bootstrap 95% CI
Index of moderated mediation			
IMM index	−0.068	0.030	[−0.133, −0.016]
Conditional indirect effects			
Female (gender = 0)	0.105	0.032	[0.047, 0.173]
Male (gender = 1)	0.038	0.021	[0.001, 0.083]
Model fit			
*R*^2^ (GSI equation)	0.171	—	—
*R*^2^ (MSE equation)	0.244	—	—
Female/male indirect ratio = 2.80× | *a*-path: female = −0.455, male = −0.162			

Bootstrap resamples = 5,000. Gender was coded 0 = female and 1 = male. The negative conditional indirect-association index therefore indicates a stronger indirect association among female students. The female indirect effect was approximately 2.80× the male indirect effect in this sample.

## 4 Discussion

### 4.1 Gendered role assignment and contextual perceptions in extracurricular music settings

The descriptive findings of the present study indicate that gendered disparities in perceived role assignment, teacher behavior, peer attitudes, and psychological outcomes were evident in this extracurricular music sample and were substantial in magnitude. Female participants reported significantly lower perceived role assignment, teacher behavior perception, and peer attitude perception than male participants, with effect sizes ranging from medium to large (*d* = −0.39 to −0.71). These patterns are consistent with prior research documenting the gendered distribution of performance opportunities across music education contexts spanning multiple countries and institutional levels (Hallam et al., 2008, 2020; Borgström Källén and Ferm Almqvist, 2025; Werner and Kuusi, 2025). What the present study adds is evidence that such disparities are also visible in extracurricular settings framed as voluntary and interest-driven. This contextual framing is theoretically relevant because the language of free choice may make patterned inequalities harder for participants to identify as structural rather than personal (Kuuse and Westerlund, 2022). At the same time, the present design does not permit the stronger claim that extracurricular contexts necessarily amplify these processes relative to formal classroom settings; that comparison remains for future work. The descriptive results therefore support treating extracurricular music classes as an important site for studying hidden-curriculum dynamics without assuming that this site is uniquely powerful on the basis of the current evidence alone.

A further implication of the present findings concerns the role of teacher and peer signals as potentially related components of a gendered learning ecology rather than fully independent sources of influence. The moderate correlation between TBP and PAP (*r* = 0.43) suggests that teacher and peer attitudes toward gender and role assignment may cluster together in students’ perceptions. However, the weaker associations involving PAP indicate that peer climate was less central than teacher-related perceptions in the focal explanatory model. This asymmetry matters. It suggests that the present data provide firmer support for attending to teacher-linked allocation and feedback processes than for making strong claims about a fully integrated teacher-peer climate mechanism. The teacher-behavior findings are therefore best read as contextual evidence that classroom fairness perceptions may shape how role opportunities are experienced, rather than as definitive proof of a separate teacher-driven internalization pathway. In the present analyses, peer attitude perception was retained as descriptive contextual evidence rather than incorporated into the core indirect-association model. This decision kept the focal model aligned with the proposed teacher-role allocation-schema-efficacy pathway, but it also means that the role of peer climate remains underdeveloped. Future models should test whether perceived peer attitudes operate as an additional upstream contextual correlate, a moderator of role-assignment interpretations, or an independent pathway linking hidden curriculum processes to self-efficacy.

### 4.2 Interpreting the indirect-association pattern

The indirect-association findings reported in this study are useful not because they establish a definitive psychological mechanism, but because they move beyond documenting mean gender differences and instead test whether those differences are statistically consistent with a plausible internalization-related association pattern. The simple indirect-association results (H3) showed that gender schema internalization statistically accounted for 21.2% of the total association between perceived role assignment (PRA) and musical self-efficacy (MSE), with a significant indirect effect [*B* = 0.098, 95% CI (0.051, 0.152)]. This pattern of partial rather than complete statistical accounting is compatible with gender schema theory (Bem, 1981), which would not imply that internalization fully explains the association between environmental role signals and self-evaluation, but rather suggests that it may be one meaningful cognitive pathway through which structural inequity becomes psychologically relevant. The retained direct association (*c*′ = 0.362) further suggests that perceived role assignment may also be linked to musical self-efficacy through additional processes not directly captured in the present model, potentially including differences in mastery experiences, performance history, evaluative feedback, or actual opportunity structures. In this respect, the findings are also broadly in line with Bandura’s (1997) account of self-efficacy development and with McCormick and McPherson’s (2003) emphasis on performance-related experiences as an important basis of efficacy belief. The supplementary serial indirect-association findings (H4) suggest a more limited and more specific interpretation than the earlier draft of the manuscript implied. The strongest indirect association from perceived teacher behavior to self-efficacy operated through perceived role assignment (*B* = 0.102), and the sequential TBP-PRA-GSI-MSE path was also significant (*B* = 0.028). By contrast, the direct TBP-GSI-MSE path was weak (*B* = 0.007) and its bootstrap interval included zero. Accordingly, the present data are more consistent with the view that teacher-related fairness perceptions matter chiefly because they are linked to how students read their role opportunities, with gender schema internalization providing a secondary elaboration of that association. Taken together, the indirect-association findings therefore support a restrained conclusion: the data are consistent with a model in which gendered role experiences are associated with self-evaluative beliefs partly through internalized gender-role schemas, but they do not by themselves identify temporal sequence or causal process.

### 4.3 Gender as a boundary condition

The conditional indirect-association findings are theoretically interesting, but their interpretation requires careful distinction between two related yet separable questions: whether gender moderates the direct association between perceived role assignment and musical self-efficacy, and whether gender moderates the indirect association operating through gender schema internalization. In the present data, the exploratory test of moderation on the direct pathway (H5) was not supported (Delta *R*^2^ = 0.002, *p* = 0.438), whereas the conditional indirect-association hypothesis (H6) was supported. Considered together, these findings suggest that gender differences were more evident in the indirect association than in the direct PRA-MSE slope. Specifically, the absence of a significant Gender x PRA interaction indicates that the positive association between perceived role assignment and musical self-efficacy did not differ significantly by gender in this sample, as both female and male participants showed significant positive slopes. By contrast, the indirect pathway through gender schema internalization was stronger among female students than among male students, with the a-path estimate linking PRA to GSI being larger in magnitude for female participants (*B* = −0.455) than for male participants (*B* = −0.162), and the conditional indirect effect being approximately 2.80 times larger among female participants. This pattern is consistent with the possibility that female students may be more likely than male students to interpret role-assignment cues in self-referential ways within extracurricular music settings. At the same time, because the present study is based on cross-sectional self-report data, the findings should not be taken as definitive evidence that female and male students process identical structural conditions through fundamentally different causal systems. A more cautious conclusion is that the strength of the internalization-related association was gender-differentiated in this sample, which provides a useful starting point for future longitudinal and multi-source research.

### 4.4 Limitations, implications, and future directions

Several limitations should be acknowledged. First, the cross-sectional design still limits causal inference: although the indirect-association models are consistent with the theorized directional sequence, they cannot establish temporal order and cannot rule out reverse or reciprocal relationships, such as lower musical self-efficacy shaping students’ perceptions of role assignment rather than the reverse. Second, all variables were assessed through same-occasion self-report measures. Although Harman’s single-factor test and the single-factor CFA suggested that common method variance was unlikely to fully explain the findings, multi-source or temporally separated data would provide stronger evidence. Third, instrument type and years of study differed across gender groups; however, robustness checks showed that the main paths, indirect effects, and conditional indirect effects remained substantively unchanged after these variables were controlled. Fourth, PRA and MSE are conceptually adjacent, but HTMT evidence and the two-factor CFA comparison supported their empirical distinction. Fifth, the non-binary/gender-diverse subgroup was too small for inferential analysis. Although exclusion from inferential models was statistically understandable, it limits the inclusiveness of the gender-related conclusions. Accordingly, the observed indirect associations should still be interpreted as theory-consistent association patterns rather than as evidence of a confirmed causal pathway.

Despite these limitations, the study carries practical implications that should be framed cautiously. The findings suggest that gender schema internalization may account for part, but not all, of the association between perceived role assignment and musical self-efficacy. Thus, interventions should not focus only on students’ beliefs; they should also address the visible allocation of musical opportunities, the feedback students receive, and the peer climate in which role assignments are interpreted. Of the contextual predictors examined, perceived teacher behavior remains the factor most plausibly amenable to intervention, even though the current data do not permit strong causal attribution. More deliberate redistribution of lead performance roles, including systematic rotation across students irrespective of gender and the explicit decoupling of musical leadership from gender-congruent expectations, may help weaken the association between role assignment inequity and self-limiting belief formation. Other mechanisms, such as prior performance success, teacher feedback, peer recognition, family support, and actual musical achievement, should also be considered when designing intervention studies. These implications should therefore be treated as provisional until evaluated with longitudinal, multi-source, or intervention-based designs.

Future research may proceed in several directions. First, longitudinal studies are needed to examine whether repeated exposure to gendered role assignment is followed by gradual strengthening of gender schema internalization over time. Second, cross-contextual comparisons across formal, extracurricular, and conservatory settings would help clarify whether the force of the hidden curriculum varies according to levels of institutional visibility and accountability. Third, future models should incorporate richer performance-history indicators, such as prior performance success, ensemble level, audition outcomes, and teacher-rated achievement, so that gendered role allocation can be evaluated against even more demanding alternative explanations. Fourth, future studies should use procedural strategies to reduce common method concerns, including multi-source reports, temporal separation, marker variables, or common latent factor approaches. Fifth, more inclusive designs are needed to represent non-binary and gender-diverse students through targeted recruitment and analytic plans that do not require excluding them from inferential analyses. Sixth, intervention-based studies are needed to test whether teacher-directed role redistribution and peer-climate change can reduce gender schema internalization and enhance musical self-efficacy among students who have been less frequently assigned visible musical roles.

## 5 Conclusion

The present study examined whether perceived gendered role assignment in extracurricular music settings was associated with musical self-efficacy, and further tested whether gender schema internalization statistically accounted for part of that association. The findings showed that female students reported lower perceived role assignment, less equitable teacher and peer climate perceptions, higher gender schema internalization, and lower musical self-efficacy and sense of agency than male students. The indirect-association analyses suggested that perceived role assignment was linked to musical self-efficacy partly through gender schema internalization, and that this indirect association was stronger among female students. These results are consistent with the proposed hidden-curriculum and gender-schema framework, but they should not be interpreted as evidence of a confirmed causal indirect process.

Taken together, the findings suggest that extracurricular music classes are a useful context for examining how perceived gendered role assignment may become linked to self-evaluative beliefs through hidden-curriculum-related processes. Addressing gender disparities in musical self-efficacy and agency may therefore require attention not only to individual motivation, but also to classroom role allocation practices, teacher feedback, peer norms, and the interpretive climates surrounding them. At the same time, because the present study relies on cross-sectional self-report data, the conclusions should be interpreted with appropriate caution. However, the revised analyses strengthen confidence in the gender-related findings by establishing configural, metric, and scalar measurement invariance across gender, showing that results remained substantively unchanged after controlling for instrument type and years of musical study, indicating that common method variance was unlikely to dominate the findings, and supporting discriminant validity between PRA and MSE. Future research using longitudinal, multi-source, and intervention-based designs will be necessary to clarify temporal direction and to assess whether more equitable role assignment practices can reduce gender schema internalization and support the development of musical self-efficacy.

## Data Availability

The original contributions presented in the study are included in the article/supplementary material, further inquiries can be directed to the corresponding author.
